# The Association Between Lymph Node Stage and Clinical Prognosis in Thyroid Cancer

**DOI:** 10.3389/fendo.2020.00090

**Published:** 2020-02-27

**Authors:** Junyi Zhang, Xiaoyun Cheng, Lei Shen, Xingchun Wang, Lu Wang, Xiaoting Sun, Shen Qu

**Affiliations:** ^1^Department of Endocrinology and Metabolism, Shanghai Tenth People's Hospital, School of Medicine Tongji University, Shanghai, China; ^2^Nanjing Medical University, Nanjing, China; ^3^Shanghai Center of Thyroid Disease, Shanghai, China; ^4^Department of Endocrinology and Metabolism, The Shanghai Tenth People's Hospital Chongming Branch, Tongji University School of Medicine, Shanghai, China; ^5^Department of Thyroid Breast Oncology, Shanghai East Hospital, Tongji University School of Medicine, Shanghai, China; ^6^The Shanghai Tenth People's Hospital, Tongji University School of Medicine, Shanghai, China

**Keywords:** thyroid cancer, lymph nodes, NX, initial distant metastasis, SEER

## Abstract

**Purpose:** To investigate the association between lymph node (N) stage and clinical outcome in thyroid cancer patients with initial distant metastasis.

**Methods:** A total of 3,198 cases (1,435 males and 1,763 females) between 2004 and 2015 with initial distant metastasis were obtained from the surveillance, epidemiology, and end results (SEER) database. Patients with a median follow up time of 13 months and a median age of 66 years were analyzed. A total of 1,407 cases had detailed information regarding the four most common metastatic organs after the year 2010. Kaplan-Meier (KM) analyses, log-rank tests, Cox regression, and logistic regression analyses were used.

**Results:** Among the whole cohort, 33.4% (1,069/3,198), 14.5% (464/3,198), 10.1% (322/3,198), 34.2% (1,094/3,198), and 7.8% (249/3,198) of the patients were at the stage of N0, NX, N1a, N1b, and N1NOS (referring to metastasis to regional lymph nodes but not otherwise specified), respectively. The KM curves demonstrated that the patients at the NX stage had the worst survival. The NX and N1b groups had the highest hazard ratios (HRs) of 1.83 (95%CI 1.46-2.31) and 1.78 (95%CI 1.52-2.10) after adjusting age, race, gender, and tumor size (*p* < 0.001) compared with N0 group. The lung was the most common metastatic site, with a rate of 51.2% (720/1,407). Compared with the N0 group, N1 patients had higher odds (OR 1.63, 95%CI 1.31-2.01, *p* < 0.001) for lung metastasis. Similar results were obtained in papillary thyroid cancer (PTC) sub-cohort.

**Conclusions:** Overall, the TC patients at the NX stage had the highest mortality risk, followed by N1b, N1a, and N0 groups. Compared with N0 patients, N1 patients were more likely to have lung metastasis. The poor prognosis for TC patients with the NX stage may make more aggressive treatment reasonable.

## Introduction

The incidence of thyroid cancer (TC) has continued to rise in the United States over the last four decades (1), and TC was reported to be the fifth most common malignant tumor in women in 2018 (2). Based on the recent data, the American Cancer Society has predicted that the number of new TC cases and deaths due to TC will become the sixth most common cause of malignant tumors for women in 2019 (3). Among several variants of TC, papillary thyroid cancer (PTC) takes up 85-90% of all cases (4).

PTC and follicular thyroid cancer (FTC) are classified as differentiated TCs that both arise from thyroid follicular cells and generally have excellent prognoses, while the poorly differentiated thyroid cancers have a poorer prognosis which probably arises from either PTCs or FTCs. Medullary thyroid cancer (MTC) has been derived from the parafollicular C cells, accounting for about 1-2% of TCs (5). Anaplastic thyroid cancer (ATC) originates from follicular cells and is an aggressive, undifferentiated, and rapidly fatal tumor (6).

The American Joint Committee on Cancer (AJCC) Staging Manual 6th Edition (7) sets out the lymph node (N) stages of cancer as follows: no metastatic nodes (N0); nodes not assessed at surgery (NX); metastases to level VI [pretracheal, paratracheal, and prelaryngeal/Delphian lymph nodes] (N1a); metastasis to unilateral, bilateral, or contralateral cervical or superior mediastinal mode metastases (N1b). In the 8th edition, lymph node stage of N0 were divided into 2 groups with more detailed information, which referred to N0a (One or more cytologic or histologically confirmed benign lymph node) and N0b (no radiologic or clinical evidence of local regional lymph node metastasis). However, the N1a stage in the 8th edition, which can be unilateral or bilateral disease, covers not only the lymph node VI but also the lymph node VII (the upper mediastinal lymph nodes). Additionally, in the 8th edition, the N1b stage referred to metastasis to levels I, II, III, IV, or V (unilateral, bilateral, or contralateral lateral neck lymph nodes) or retropharyngeal lymph nodes, which include all disease in the lateral neck (8). Compared with 6th edition, the other N stages of 8th edition remain unchanged. In the United States, it has been reported that 5% of TC patients have distant metastasis (9), while the most commonly observed metastatic sites have been reported as lung and bone, followed by brain and liver (10).

Although previous studies have shown that TC patients have favorable survival outcomes, with a mortality rate of ~10% and a propensity to have lymph node metastases (11, 12), certain lymph node involvement was reported to have an adverse impact on the prognosis for TC (13). Moreover, the prognosis for NX patients and the association between N stage and distant metastasis remain unknown. In addition, most of these studies were based on data derived from a small sample size population and were limited due to the types of statistical analyses undertaken.

The objective of this study is to investigate the prognosis of TC patients at the NX stage and to determine the association between N stages and distant metastasis in TC based on the surveillance, epidemiology, and end results (SEER) database.

## Materials and Methods

### Patients and Clinical-Pathological Data

The purpose of this retrospective study is to investigate the association between N stage and distant metastasis, and disease-specific mortality of TC and its common variants, including PTC, FTC, ATC, and MTC. Data on TC patients were retrieved from the SEER database (https://seer.cancer.gov/), which is maintained by the National Cancer Institute. The SEER project is a United States population-based cancer registry that began in 1973 and includes ~34.6% of the US population.

Patients with TC were classified by the 3rd Edition of the International Classification of Diseases for Oncology (ICD-O-3). Lymph node stages were classified into five groups according to the 6th AJCC Staging Manual (7) and SEER as following: N0, NX, N1a, N1b, and N1NOS, in which N1NOS referred to metastasis to regional lymph nodes but not otherwise specified. These groups were analyzed to determine the association between the N stage and the specific mortality of TC and its common variants. In addition, patients with detailed information on the four most common organ metastases after the year 2010 were identified to determine the association between N stage and distant metastasis.

### Statistical Analyses

Survival probabilities for TC and its common variants were assessed using Kaplan-Meier (KM) analyses and log-rank tests to compare the differences between Kaplan-Meier curves of different lymph node groups. Cox regression was performed to calculate the hazard ratios (HRs) and 95% confidence intervals (CIs) to determine the effects of different N stages on disease-specific mortality in TC and its common variants. The association between N stages and distant organ metastases were analyzed by logistic regression. A two-tailed *P* < 0.05 was considered to be statistically significant. The Statistical Package for Social Science version 25.0 (SPSS, Inc., New York, NY, USA) was used for data analyses.

## Results

### Patient Characteristics

A total of 3,198 TC patients (1,435 males and 1,763 females) with initial distant metastasis between 2004 and 2015 were investigated. The median follow-up time was 13 months (interquartile range [IQR], 2-46 months) and the median age was 66 years (IQR, 55-76 years). In addition, 1,407 patients with detailed information concerning the four most common organ metastases after the year 2010 were identified. The clinical characteristics of the whole cohort were shown in Table 1.

**Table 1:** The clinical characteristics of thyroid cancer patients with different lymph node stage.

**Characteristics**		**N0**		**NX**		**N1a**		**N1b**		**N1NOS**	
**number**		**1,069**		**464**		**322**		**1,094**		**249**	
		**Number**	**%**	**Number**	**%**	**Number**	**%**	**Number**	**%**	**Number**	**%**
Age	<55	217	20.2	53	11.4	91	28.2	355	32.4	71	28.5
	>55	852	79.7	411	88.6	231	71.7	739	67.6	178	71.5
Gender	Male	405	37.8	182	39.2	145	45	570	52.1	133	53.4
	Female	664	62.1	282	60.8	177	55	524	47.9	116	46.6
Race	White	733	68.6	319	68.8	255	79.2	888	81.2	188	75.5
	Black	151	14.1	66	14.2	29	9	71	6.5	20	8
	Others	183	17.1	75	16.2	37	11.5	132	12.1	41	16.5
	Unknown	2	0.2	4	0.9	1	0.3	3	0.3	-	0
Tumor size	TX	155	14.5	239	51.5	20	6.2	96	8.8	29	11.6
	T0	49	4.6	15	3.2	3	0.9	10	0.9	3	1.2
	T1	144	13.5	7	1.5	26	8.1	57	5.2	14	5.6
	T2	141	13.2	19	4.1	28	8.7	51	4.7	7	2.8
	T3	238	22.3	29	6.3	95	29.5	264	24.1	47	18.9
	T4a	96	9	33	7.1	52	16.1	208	19	44	4.4
	T4b	228	21.3	92	19.8	93	28.9	370	33.8	89	35.7
	T4NOS	18	1.7	30	6.5	5	1.6	38	3.5	16	6.4
Extrathyroid extension	No	548	51.3	81	17.5	105	32.6	295	27	68	27.3
	Yes	402	37.6	127	27.4	198	61.5	700	64	146	58.6
	Unknown	119	11.1	256	55.2	19	5.9	99	9	35	14.1
Thyroid cancer specific death	Alive	443	41.4	139	30	144	44.7	426	38.9	99	39.8
	Death	388	36.3	223	48.1	124	38.5	480	43.9	115	46.2
	Unknown	238	22.3	102	22	54	16.8	188	17.2	35	14.1
Married	Single (never married)	169	15.9	74	15.9	50	15.5	228	20.8	53	21.3
	Separated	13	1.2	4	0.8	-	0	16	1.5	2	0.8
	Married (including common law)	549	51.4	206	44.4	190	59	608	55.6	123	49.4
	Widowed	202	18.9	111	23.9	49	15.2	135	12.3	30	12
	Divorced	90	8.4	32	6.9	22	6.8	71	6.5	27	10.8
	Unmarried or Domestic Partner	1	0	1	0.2	1	0.3	1	0	2	0.8
	Unknown	45	4.2	36	7.8	10	3.1	35	3.2	12	4.8

*According to the American Joint Committee on Cancer (AJCC) Staging Manual 6th Edition, tumor size was classified into 8 groups as follows: Primary tumor cannot be assessed (TX); No evidence of primary tumor (T0); Tumor 2 cm or less in greatest dimension limited to the thyroid (T1); Tumor more than 2 cm but not more than 4cm in greatest dimension limited to the thyroid (T2); Primary tumor diameter >4 cm limited to the thyroid or with minimal extrathyroidal extension (T3); Tumor of any size extending beyond the thyroid capsule to invade subcutaneous soft tissues, larynx, trachea, esophagus, or recurrent laryngeal nerve (T4a); Tumor invades prevertebral fascia or encases carotid artery or mediastinal vessels (T4b); Extrathyroidal invasion but not otherwise specified (T4NOS)*.

Table 2 shows that N1b was the most common N stage for patients with TC in the whole cohort. The incident rate was 33.4% (1,069/3,198), 14.5% (464/3,198), 10.1% (322/3,198), 34.2% (1,094/3,198), and 7.8% (249/3,198) for N0, NX, N1a, N1b, and N1NOS stage, respectively. The lung metastatic rate was 51.2% (720/1,407), and the bone metastatic rate was 19.3% (271/1,407). Among all variants of TC, PTC was the most common type (*n* = 1,475 patients), and the incidence rate was 31.9% (471/1,475), 8.8% (130/1,475), 13.1% (193/1,475), 38.4% (566/1,475), and 7.8% (115/1,475) for PTC patients at N0, NX, N1a, N1b, and N1NOS stage, respectively. The metastatic rates for bone and lung among patients were 61.8% (379/613) and 19.1% (117/613), respectively. Also, the incidence rate of each N stage and the metastatic rates for patients with other common variants of TC, including FTC, ATC, and MTC were shown in Table 2.

**Table 2:** The general information of lymph node stage and distant metastasis in thyroid cancer and its common variants.

**Characteristics**	**TC**		**PTC**		**FTC**		**ATC**		**MTC**	
**number**	**3,198**		**1,475**		**409**		**458**		**226**	
	**Number**	**%**	**Number**	**%**	**Number**	**%**	**Number**	**%**	**Number**	**%**
N0	1,069	33.4	471	31.9	238	58.2	114	24.9	38	16.8
NX	464	14.5	130	8.8	90	22.0	65	14.2	27	11.9
N1a	322	10.1	193	13.1	23	5.6	44	9.6	18	8.0
N1b	1,094	34.2	566	38.4	45	11.0	186	40.6	126	55.8
N1NOS	249	7.8	115	7.8	13	3.2	49	10.7	17	7.5
Number^a^	1,407		613		186		236		106	
Bone metastasis	271	19.3	117	19.1	73	39.2	16	6.8	18	17.0
Lung metastasis	720	51.2	379	61.8	49	26.3	148	62.7	16	15.1

PTC papillary thyroid cancer, FTC follicular thyroid cancer; ATC anaplastic thyroid cancer, MTC medullary thyroid cancer.

a 1,407 cases had information of detailed organ metastasis.

### Comparison of Disease-Specific Mortality Between Different N Stage in TC and Its Common Variants

The mortality rate was 46.7% (388/831), 61.6% (223/362), 46.3% (124/268), 53.0% (480/906), and 53.7% (115/214) for N0, NX, N1a, N1b, and N1NOS, respectively. Compared with N0 patients, the crude hazard ratio (HR) of disease-specific mortality was 1.71 (95% CI 1.45-2.02, *P* < 0.001), 0.98 (95%CI 0.80-1.19, *P* = 0.808), 0.74 (95%CI 0.65-0.85, *P* < 0.001), and 0.81 (95%CI 0.65-1.00, *P* = 0.045) for NX, N1a, N1b, and N1NOS patients, respectively. After adjusting age, race, sex, and tumor size, the adjusted HRs of NX, N1b, and N1NOS groups remained significant, was 1.83 (95% CI 1.46-2.31, *P* < 0.001), 1.78 (95% CI 1.52-2.10, *P* < 0.001), and 1.46 (95% CI 1.14-1.86, *P* = 0.003), respectively. In PTC, the mortality rate was 31.4% (113/360), 45.4% (44/97), 28.5% (45/158), 31.8% (149/468), and 27.0% (27/100) for N0, NX, N1a, N1b, and N1NOS group, respectively. Compared with N0 PTC group, the crude HR was 1.68 (95% CI 1.19-2.38, *P* = 0.004) and 1.02 (95% CI 0.80-1.31, *P* = 0.857), and the adjusted HR was 1.83 (95% CI 1.10–3.02, *P* = 0.019) and 1.70 (95% CI 1.27-2.29, *P* < 0.001) for NX and N1b group, respectively. In FTC, N1NOS group has the highest mortality rate of 70.0% (7/10), while NX group has the lowest mortality rate of 36.8% (28/76). Compared with N0 FTC patients, the hazards of NX and N1NOS groups did not show any significance. N1b group had a higher crude HR but the significance was lost after adjustment. N1a group had a crude HR of 2.06 (95% CI 1.09–3.89, *P* = 0.026) and an adjusted HR of 2.33 (95% CI 1.17–4.65, *P* = 0.016). In ATC, the mortality rates were all over 80.0% for all N stages. NX ATC group had the highest mortality rate of 96.0% (48/50), followed by 93.3% (83/89) for N0 group, 88.1% (37/42) for N1NOS group, 86% (135/157) for N1b group and 85.0% (34/40) for N1a group (Table 3).

**Table 3:** The Association between lymph node stage and thyroid cancer specific mortality. (SEER database years of 2004-2015).

**Group**	**Mortality**	**Unadjusted**		**Adjusted**^**a**^	
	***n*/*N* (%)**	**HR (95%CI)**	***P***	**HR (95%CI)**	***P***
**TC**					
N0	388/831 (46.7)	Ref.			
NX	223/362 (61.6)	1.71 (1.45-2.02)	<0.001	1.83 (1.46-2.31)	<0.001
N1a	124/268 (46.3)	0.98 (0.80-1.19)	0.808	1.07 (0.84-1.35)	0.601
N1b	480/906 (53.0)	0.74 (0.65-0.85)	<0.001	1.78 (1.52-2.10)	<0.001
N1NOS	115/214 (53.7)	0.81 (0.65-1.00)	0.045	1.46 (1.14-1.86)	0.003
**PTC**					
N0	113/360 (31.4)	Ref.			
NX	44/97 (45.4)	1.68 (1.19-2.38)	0.004	1.83 (1.10-3.02)	0.019
N1a	45/158 (28.5)	0.90 (0.64-1.27)	0.557	1.04 (0.69-1.56)	0.867
N1b	149/468 (31.8)	1.02 (0.80-1.31)	0.857	1.70 (1.27-2.29)	<0.001
N1NOS	27/100 (27.0)	0.72 (0.47-1.11)	0.137	0.85 (0.51-1.41)	0.521
**FTC**					
N0	73/194 (37.6)	Ref.			
NX	28/76 (36.8)	1.21 (0.77-1.88)	0.406	1.02 (0.53-1.96)	0.956
N1a	11/17 (64.7)	2.06 (1.09-3.89)	0.026	2.33 (1.17-4.65)	0.016
N1b	22/38 (57.9)	2.09 (1.29-3.38)	0.003	1.80 (0.98-3.31)	0.057
N1NOS	7/10 (70.0)	2.15 (0.99-4.70)	0.055	1.95 (0.70-5.48)	0.204
**ATC**					
N0	83/89 (93.3)	Ref.			
NX	48/50 (96.0)	1.25 (0.87-1.80)	0.219	1.22 (0.79-1.89)	0.374
N1a	34/40 (85.0)	0.86 (0.58-1.28)	0.455	0.85 (0.51-1.40)	0.510
N1b	135/157 (86.0)	1.08 (0.82-1.42)	0.599	1.25 (0.91-1.73)	0.171
N1NOS	37/42 (88.1)	1.10 (0.75-1.63)	0.622	1.11 (0.69-1.77)	0.670

TC, thyroid cancer; PTC, papillary thyroid cancer; FTC, follicular thyroid cancer; ATC, anaplastic thyroid cancer; HR, hazard ratios; CI, confidence interval.

a Adjusted for age, race, gender, and tumor size.

### Comparison of the Lung and Bone Metastasis in TC and Its Common Variants

In TC, the metastatic rate of bone was 25.9% (151/584), 21.9% (39/178), and 8.9% (81/910) for N0, NX, and N1 group, respectively. Compared with the N0 group, the NX group did not show any difference in the odds of bone metastasis. However, the crude OR of the N1 group was 0.46 (95%CI 0.37-0.57, *P* < 0.001) and remained significant after adjustment. The metastatic rate of the lung was 37.5% (219/584), 36.0% (64/178), and 48.0% (437/910) for N0, NX, and N1 group, respectively. Compared with the N0 group, N1 patients had higher odds of lung metastasis, with crude OR of 1.63 (95% CI 1.31-2.01, *P* < 0.001) and an adjusted OR of 1.99 (95% CI 1.55-2.55, *P* < 0.001). NX group did not show significant OR because of the small sample size (Table 4).

**Table 4:** The association between lymph node stage and distant metastasis in thyroid cancer (SEER database years of 2010-2015).

**Group**			**Metastatic site rate**	**Unadjusted**		**Adjusted**^**a**^	
			***n*/*N* (%)**	**OR (95%CI)**	***P***	**OR (95%CI)**	***P***
**TC**							
	Bone alone		271/1,407 (19.3)				
		N0	151/584 (25.9)	Ref.			
		NX	39/178 (21.9)	0.89 (0.63-1.25)	0.491	0.77 (0.42-1.21)	0.210
		N1	81/910 (8.9)	0.46 (0.37-0.57)	<0.001	0.44 (0.34-0.57)	<0.001
	Lung alone		720/1,407 (51.2)				
		N0	219/584 (37.5)	Ref.			
		NX	64/178 (36.0)	1.01 (0.72-1.41)	0.979	1.53 (0.89-2.65)	0.126
		N1	437/910 (48.0)	1.63 (1.31-2.01)	<0.001	1.99 (1.55-2.55)	<0.001
**PTC**							
	Bone alone		117/613 (19.1)				
		N0	63/258 (24.4)	Ref.			
		NX	10/53 (18.7)	0.72 (0.38-1.38)	0.322	0.56 (0.17-1.79)	0.326
		N1	44/480 (9.2)	0.41 (0.29-0.58)	<0.001	0.42 (0.29-0.62)	<0.001
	Lung alone		379/613 (61.8)				
		N0	99/258 (38.4)	Ref.			
		NX	21/53 (39.6)	1.12 (0.62-2.02)	0.712	1.15 (0.41-3.21)	0.793
		N1	259/480 (54.0)	1.70 (1.25-2.32)	0.001	2.22 (1.56-3.16)	<0.001

TC, thyroid cancer; PTC, papillary thyroid cancer; OR, odd ratio; CI, confidence interval.

a Adjusted for age, race, gender, and tumor size.

Similar results could be obtained in PTC. The metastatic rate of bone was 24.2% (63/258), 18.7% (10/53), and 9.2% (44/480) for N0, NX, and N1, respectively. Compared with the N0 group, the N1 group showed significant OR of 0.41 (95%CI 0.29-0.58, *P* < 0.001) and remained significant after adjustment. The metastatic rate of the lung was 38.4% (99/258), 39.6% (21/53), and 54.0% (259/480) for N0, NX, and N1, respectively. Compared with the N0 group, N1 patients had significant crude and adjusted OR of 1.70 (95%CI 1.25-2.32, *P* = 0.001) and 2.22 (95%CI 1.56-3.16, *P* < 0.001) (Table 4).

### Kaplan–Meier Analyses of Disease-Specific Survival of TC Patients and Its Variants

**Figure 1:**
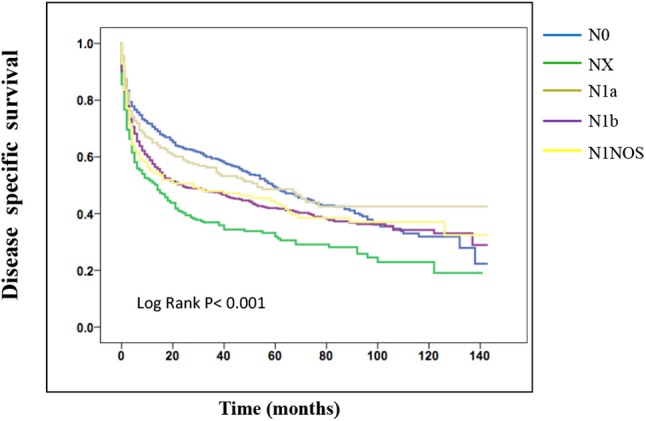
Disease specific survival of TC patients stratified by N stage using Kaplan-Meier analysis.

**Figure 2:**
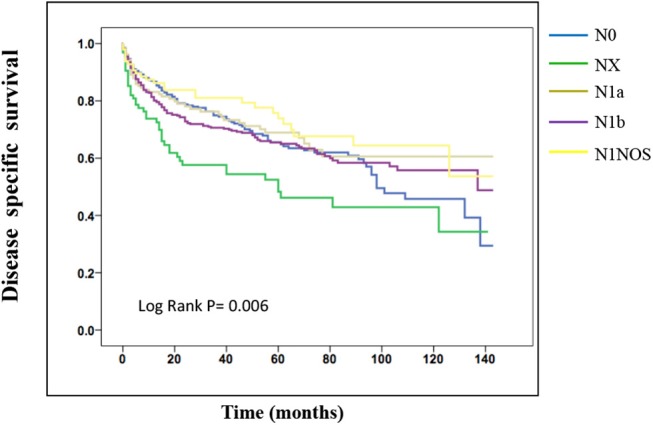
Disease specific survival of PTC patients stratified by N stage using Kaplan-Meier analysis.

**Figure 3:**
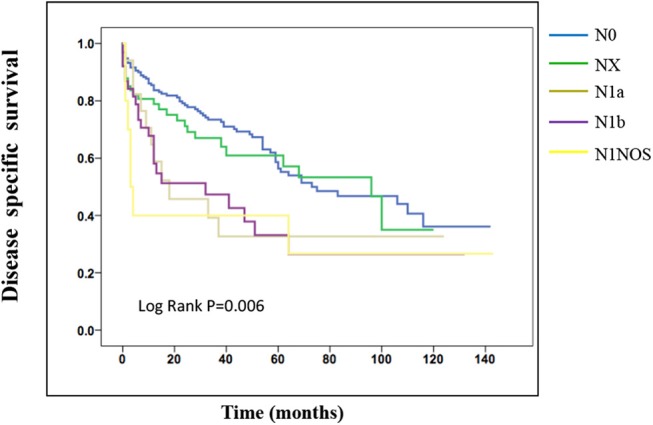
Disease specific survival of FTC patients stratified by N stage using Kaplan-Meier analysis.

**Figure 4:**
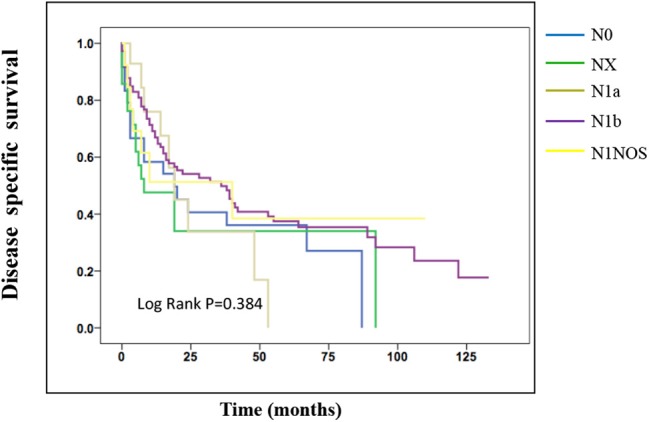
Disease specific survival of MTC patients stratified by N stage using Kaplan-Meier analysis.

**Figure 5:**
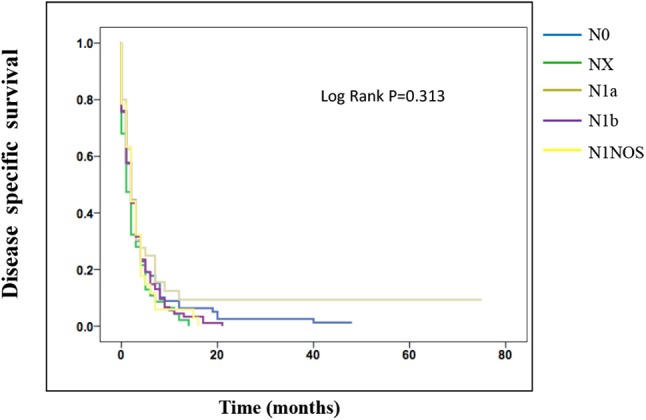
Disease specific survival of ATC patients stratified by N stage using Kaplan-Meier analysis.

On the analysis of disease-specific survival of all TC patients, NX was associated with a statistically significant decrease in survival, followed by N1NOS and N1b groups, while N1a and N0 showed better survival (log-rank *P* < 0.001) (Figure 1). In PTC, The NX group had the poorest survival with a sharp decrease, followed by N1b, N1a, and N0 groups, while N1NOS groups had better survival (log-rank *P* = 0.006) (Figure 2). In FTC, the worst survival was obtained in the N1NOS group, followed by N1a and N1b groups with a sharp decrease, while N0 and NX groups had better survival (log-rank *P* = 0.006) (Figure 3). There was no statistically significant decrease in MTC (Figure 4). In ATC, the N stage had no significant effect on disease-specific survival (log Rank *P* = 0.313) (Figure 5).

## Discussion

In this study, we have investigated the association between the N stage and disease-specific mortality in TC patients with initial distant metastasis. Our data indicated that TC patients with NX had the highest mortality risk, while the N0 group had the lowest mortality risk. In addition to this, our results also suggest that the N1 group had a higher probability of lung metastasis.

The TNM stage system was designed empirically to predict the survival of most TC patients and to guide therapeutic methods and clinical follow-up setup. An interesting retrospective study by Adam et al. found that the cervical lymph node metastases were not in correlation with a favorable but compromised prognosis in PTC patients younger than 45 years (14). Compared with the 7th AJCC Staging Manual, the 8th AJCC Staging Manual has a remarkable change in the definition of N1a and N1b, which may result in better accuracy of central and lateral neck disease (8). However, there is no special attention paid to the NX stage. Previous studies have investigated the risk factors and clinical outcomes of the NX stage in TC and showed its association with poor prognosis (15–17). A study by Baek et al. retrospectively analyzed data concerning 189 PTC patients who had undergone surgery and confirmed that lymph node metastasis was a factor for increasing the risk of recurrence (18, 19). Although it has been reported that lymph node involvement has prognostic significance for TC patients (14, 17, 20), the significance of different N stages still remains controversial. In a study conducted in 1961, TC patients whose disease extended beyond the capsule of the thyroid gland to the regional lymph nodes, surrounding organs or tissues, etc., had a much higher survival rate than that of other groups at 20 years after surgery (21). On the contrary, recent study results suggest that the lymph node involvement have a bad effect on the prognosis of TC (22, 23). Whereas in such a situation, there is still not much attention paid to the investigation on the survival outcomes of patients with different lymph node stages, especially for the NX stage. Liu et al. reported that differentiated TC patients with the NX stage had an unexpectedly poor prognosis via evaluating the survival outcomes (16). They have found that either cancer-specific mortality or all-cause mortality for NX stage patients was higher than N1 and N0 stage patients. Meanwhile, they ascertained that the prognosis for NX stage patients was worse compared to N0 patients. After propensity score matching for relevant confounding factors, patients with N1 stage sustained similar results, which may result from insufficient use of thyroidectomy. In our study, we found that the NX patients had the highest mortality followed by N1b, N1NOS, and N1a groups in PTC, which was consistent with the results of Liu et al. However, the NX did not show any prognostic value when analyzing FTC population alone. To our knowledge, previous studies have noted the risk factors of prognosis for TC patients, such as BRAF mutation (24) and multifocality (25), and categorized the disease stages using the N stage of N0-N1 to assess the clinical outcomes of TC (8). However, the studies focused on the prognosis of NX stage TC patients with distant organ metastasis at diagnosis are scarce. Our study filled the research gap by investigating the prognosis of NX in TC patients, which may provide new clinical-relevant information for improving the treatment strategies. The incidence of TC is increasing rapidly with its diagnosis becoming more and more convenient with the development of images, computed tomography, magnetic resonance imaging, and other medical auxiliary inspection technologies. Even a small percentage change in the mortality rate is likely to result in a big difference in disease-specific mortality. More attention should be given to the NX patients in terms of clinical diagnosis and treatment because of its poor prognosis compared with other N stages. Similar results could be obtained in PTC patients, which may be the consequence of the fact that PTC accounts for ~85-90% of all TC cases (4). Conversely, in the FTC group, our study did not show results similar to the whole TC group and PTC group, which indicates that the N1a was a risk factor for FTC-specific death instead of the NX and N1b. A study by Witte et al. reported that the lymph node metastasis is an important factor affecting FTC patients' survival, while no detail information such as the lymph node location was provided despite the N stage (26). Thus, it still needs further investigation on the correlation between the N stage and the prognosis of patients with FTC.

A high potential survival risk has been reported for patients with TC and distant organ metastasis (27). The four most common distant metastasis organs identified in the 2010 AJCC Staging Manual were bone, brain, liver, and lung. The previous study has shown that lungs and bones were more likely to be metastatic sites than brains and livers for TC patients (28). Additionally, organ metastases were reported to have a correlation with worse disease-specific survival in TC (29). We have investigated the association between the N stage and the two organ metastases, lung, and bone. The metastatic rate for lung is 51.2% (720/1,407), which is consistent with the results of a study by Hirsch et al., the study reported that the lung was the most common distant metastatic organ with an incidence rate of 85.5% (118/138) (28). According to the previous reports the most common cause of death for TC patients with lung metastasis to be respiratory failure due to the progression of lung metastasis (30, 31). Therefore, chest computed tomography should be used to diagnose TC patients, especially PTC patients with N1 stage. Bone is regarded as a common metastatic site. Though Slook et al. reported that bone metastasis is linked to high mortality risk as a risk factor having an impact on the survival of differentiated TC patients (32), there was no previous study focused on the association between N stage and bone metastasis. Our study indicated that N1 patients were more likely to have lung metastasis than bone metastasis. TC patients with regional lymph node metastasis should carefully undergo ultrasonography or biopsy of lymph nodes must be performed to potentially identify clinical lung metastasis rather than bone metastasis, even though bone metastasis has been reported to lead to high morbidity rates for skeletal-related events such as pathologic fractures, spinal cord compression, the requirement for bone irradiation, or bone surgery (7). In terms of treatments, even though radioactive iodine therapy has great significance in TC patients with lung metastasis, more than half of the cases showed progressive metastasis (33). For TC patients with radioactive iodine ablation-refractory metastasis, Moneke et al. (34) reported the effectiveness of surgical resection. More aggressive treatment could be arranged for individuals to balance the clinical benefits and harms and reach the optimal results.

This study had some limitations. Though the SEER database is a well-validated dataset, this was a retrospective study. Moreover, the SEER database does not provide information which may be required for further prospective studies, such as gene mutations and types of therapy. Besides, our study only obtained the data between 2004 and 2015 from the SEER database to investigate the association between N stage and distant organ metastases of lungs and bones. Future studies on the other two common distant metastases sites, brains and livers, and multi-organ metastases are being conducted.

TC Patients with NX stage had the poorest survival, and the highest disease-specific mortality risk, followed by patients with N1b, N1a, and N0 lymph node stages. The lung was found to be the most common metastatic site. Among different lymph node stages, N1 patients were more prone to lung metastasis. Therefore, we recommend chest computed tomography screening during follow-ups for TC patients, particularly PTC patients with N1 stage. Our study provided new information regarding the treatment of patients with NX stage differentiated TC.

## Data Availability Statement

Data on TC patients were retrieved from the SEER database (https://seer.cancer.gov/), which is maintained by the National Cancer Institute. The datasets generated for this study are available on request to the corresponding author.

## Author Contributions

JZ, XC, LS, and SQ: conception and design. All authors contributed to acquisition, statistical analysis or interpretation of the data, drafting of the manuscript. All authors reviewed and approved the final version of the manuscript.

### Conflict of Interest

The authors declare that the research was conducted in the absence of any commercial or financial relationships that could be construed as a potential conflict of interest.
